# Neuroinvasive Cryptococcosis in an Immunocompetent Patient with a Negative Spinal Fluid *Cryptococcus* Antigen

**DOI:** 10.1155/2015/857539

**Published:** 2015-04-14

**Authors:** Rocio C. Garcia-Santibanez, Veenu Gill, Stanley Yancovitz, DeWitt Pyburn

**Affiliations:** ^1^Department of Neurology, Mount Sinai Beth Israel, New York, NY 10003, USA; ^2^Department of Infectious Diseases, Mount Sinai Beth Israel, New York, NY 10003, USA

## Abstract

58-year-old man presented with headache, nausea, vomiting, and gait disturbance. Brain MRI showed meningeal enhancement and herniation. Serum *Cryptococcus* antigen was positive but spinal fluid antigen and cultures were negative. A cerebellar biopsy revealed nonencapsulated *Cryptococcus*. He completed antifungal therapy. Serum *Cryptococcus* antigen titer decreased. He had a full neurological recovery.

## 1. Report

A 58-year-old man with history of hypertension presented with a headache that started two months prior to admission. The headache was constant and associated with nausea and vomiting. It significantly worsened over the two weeks prior to admission, with associated lethargy, photophobia, gait disturbance, and diplopia. He visited an outside hospital a week priorly, had a lumbar puncture, was diagnosed with aseptic meningitis, and was discharged. According to his wife, he had been sleeping all day two days prior to admission. Patient denied any tobacco, alcohol, drug use, unprotected sexual encounters, sick contacts, or foreign travel. He was born in South America, worked an executive job, and at times volunteered cleaning the church. Review of systems was positive for an unintentional 15-pound weight loss.

Vital signs on admission were stable. On neurological exam patient was somnolent, in pain, holding his head, and disturbed by the questioning. He was oriented to person only but was able to name, read, and repeat. His writing was impaired. Attention and memory were also impaired. His cranial nerve exam showed bilateral papilledema and bilateral sixth nerve palsies. Motor, sensory, and reflex examinations were normal. Coordination was impaired in bilateral finger to nose and heel knee shin. His gait was widely based and was unable to tandem.

**Figure 1:**
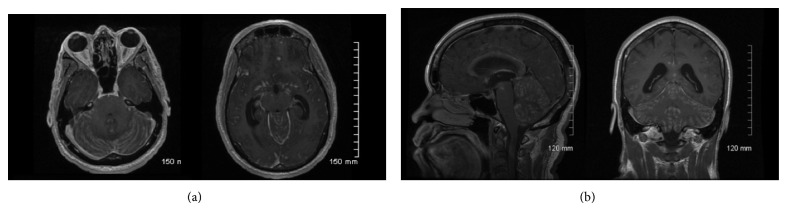
Initial MRI: T1 postcontrast axial images on the top row, sagittal and coronal on bottom row.

Initial CT examination showed mass effect in the posterior fossa, resulting in upward transtentorial and downward tonsillar herniation, along with a moderate noncommunicating hydrocephalus. Blood work in the emergency department was unremarkable including complete blood count, metabolic panel, and a negative HIV test. A serum* Cryptococcus* antigen screen was ordered in the emergency department due to the significant hydrocephalus and the recent diagnosis of meningitis. The test returned positive with a titer of 1 : 50. The patient was started on liposomal amphotericin B and flucytosine and admitted to the neurology step-down unit. A brain MRI showed enhancement of the cerebellar and cerebral meninges more extensive in the posterior fossa, downward displacement of the tonsils and the vermis, and small early subacute infarcts in the cerebellar hemispheres (Figure 1). An external ventricular drain was placed for drainage and pressure monitoring. CSF samples were collected and analyzed multiple times (Table 1).* Cryptococcus* antigen screen in the CSF and cultures, including fungal and mycobacterial, were repeatedly negative. Given the negative CSF findings, a pan CT-scan and gallium scan were done looking for an alternative explanation. Both were unremarkable. Anti-GM-CSF antibodies were not tested.

**Figure 2:**
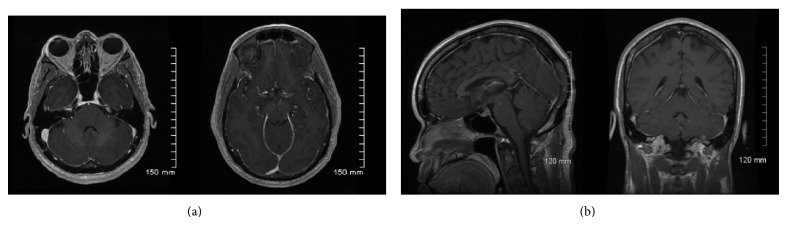
Follow-up MRI: T1 postcontrast axial images on the top row, sagittal and coronal on bottom row.

A biopsy of the cerebellum and meninges was performed. It revealed granulomatous inflammation limited to the leptomeninges. Organisms were seen in association with giant cells. The organisms were positive for GMS, PAS, and Fontana-Masson and negative for FITE, Gram, mucicarmine, and toxoplasma immunostain. The final report was granulomatous meningitis with nonencapsulated variant of* Cryptococcus*. A repeat MRI of the brain ten days into treatment showed a decrease in the diffuse abnormal signal enhancement as well as resolution of the previously seen cerebellar edema (Figure 2). The patient was discharged after twenty days with complete resolution of symptoms and complete neurological recovery. After completion of two weeks of induction, he was switched to fluconazole. He was seen at one, two, and three months as an outpatient. He remains asymptomatic and will complete ten months of fluconazole therapy. His serum* Cryptococcus* titer at two months was 1 : 10.

## 2. Discussion

Cryptococcosis is not only a disease of immunocompromised patients but is also seen in a substantial number of immunocompetent individuals [1]. We presented a case of a patient with granulomatous meningitis due to* Cryptococcus* with negative fungal cultures, negative* Cryptococcus* antigen in the CSF, and no evidence of capsule on histopathology but a positive serum* Cryptococcus* antigen. The fact that he had detectable polysaccharide in serum leads us to believe that it was indeed an encapsulated strain; however, similar findings were not observed in the spinal fluid or even on pathology. Stable mutants with a reduced capacity to produce capsule have been isolated from suspensions of* Cryptococcus neoformans* after treatment with a mutagen as described in a study by Jacobson et al. [2]. Phenotypically, they can be either acapsular (lacking the major capsular antigen) or hypocapsular (indistinguishable from the wild type). Whether there were any intrinsic immunological host factors or factors of the disease process itself that could have led to the inability to form a capsule within the brain tissue is unclear. Another study described that capsule structure modifications can occur after prolonged in vitro growth or in vivo passaging and that this could vary in different organs [3]. There is evidence that capsular rearrangements including changes in the density occur during human Cryptococcal meningitis which have significant interactions with the host immune system [4]. Changes in capsular structure can occur during passage across the blood brain barrier which not only influences the interaction between the pathogen and the host but also can affect the diagnosis [4, 5]. Studies have shown that changes in the polysaccharide structure produce false negative results, and this is what we believe might have happened in our patient [4, 6].

There has not been a similar case of cryptococcal meningitis described in the literature with one result suggesting infection with an encapsulated strain but no evidence of capsule production in the affected tissue. Through this case report, we intend to make clinicians aware of one of the challenges encountered in the diagnosis of* Cryptococcus* infections.

## Figures and Tables

**Table 1:** Serum and CSF results.

Test results	Days 1-2	Days 3-4	Days 8-9	Day 12
*CSF (from EVD) *				
Protein	<10	15	58	<10
Glucose	82	109	78	4
WBC	6	7	53	10
RBC	570	2510	107	77
*Cryptococcus* antigen	Negative			
CSF culture	Negative	Negative		
Fungal culture	Negative	Negative		
AFB culture	Negative	Negative		
EBV PCR	Negative			
ACE	5			
Cytology	Negative			
*Serum *				
HIV screen	Negative			
HIV viral load	<20			
WBC	7.6			
Hemoglobin	13.6			
Hematocrit	39.9			
Platelets	272			
Sodium	134			
Potassium	4.3			
BUN	13			
Creatinine	0.76			
LDH	325			
AST	14			
ALT	34			
Alkaline phosphatase	57			
ANA		Negative		
